# Virion Background and Efficiency of Virion Incorporation Determine Susceptibility of Simian Immunodeficiency Virus Env-Driven Viral Entry to Inhibition by IFITM Proteins

**DOI:** 10.1128/JVI.01488-16

**Published:** 2017-01-03

**Authors:** Florian Wrensch, Markus Hoffmann, Sabine Gärtner, Inga Nehlmeier, Michael Winkler, Stefan Pöhlmann

**Affiliations:** Infection Biology Unit, German Primate Center, Göttingen, Germany; University of Illinois at Chicago

**Keywords:** entry, IFITM, SIV

## Abstract

Interferon-induced transmembrane proteins (IFITMs) can inhibit the cellular entry of several enveloped viruses, including simian immunodeficiency virus (SIV). The blockade of SIV by IFITMs is isolate specific, raising the question of which parameters impact sensitivity to IFITM. We show that the virion context in which SIV-Env is presented and the efficiency of virion incorporation determine Env susceptibility to inhibition by IFITMs. Thus, determinants other than the nature of the envelope protein can impact the IFITM sensitivity of viral entry.

**IMPORTANCE** The host cell-encoded IFITM proteins can block viral entry and are an important component of the innate defenses against viral infection. However, the determinants controlling whether a virus is susceptible to blockade by IFITM proteins are incompletely understood. Our study shows that the amount of envelope proteins incorporated into virions as well as the nature of the virion particle itself can impact the sensitivity of viral entry to IFITMs. These results show for the first time that determinants other than the viral envelope protein can impact sensitivity to IFITM and have implications for the interpretation of previously published data on inhibition of viruses by IFITM proteins. Moreover, our findings might help to define the mechanism underlying the antiviral activity of IFITM proteins.

## INTRODUCTION

The interferon (IFN) system is an integral component of innate immunity and an important first-line defense against invading viruses. The IFN system is triggered by sensors that recognize pathogen-associated molecular patterns and, upon ligand binding, induce signaling cascades that trigger the production of IFN (1, 2). Binding of IFN to IFN receptors then induces the expression of IFN-stimulated genes, several of which encode proteins with antiviral activity (3). Understanding how these antiviral effectors block viral spread may allow devising novel antiviral strategies and is thus the focus of many current research efforts.

The family of IFN-induced transmembrane proteins (IFITMs) comprises five members (in humans), including the antivirally active proteins IFITM1, -2, and -3 (4). These proteins inhibit host cell entry driven by the glycoproteins of many enveloped viruses, including influenza A viruses (FLUAV), coronaviruses, and filoviruses (5–12). Expression of IFITMs blocks entry at the stage of glycoprotein-driven fusion of viral and cellular membranes, specifically during hemifusion or the formation of fusion pores (13, 14). This blockade might be due to IFITMs modifying the physical properties of cellular membranes, potentially via IFITM-IFITM interactions (15) or by altering membrane cholesterol levels (16).

The IFITM-mediated blockade of viral entry seems to be restricted largely to viruses that enter target cells via fusion with endo- or lysosomal membranes, although IFITM1 can be expressed at the cell surface (8). Thus, one would assume that entry of human immunodeficiency virus (HIV) and simian immunodeficiency virus (SIV), which is believed to proceed mainly at the plasma membrane, is not inhibited. The laboratories reporting the identification of IFITMs as antiviral factors indeed failed to detect an IFITM-dependent blockade of HIV-1 infection (4, 6). Nevertheless, subsequent studies reported that IFITMs restrict HIV and SIV entry (11, 17, 18). More recently, it was reported that IFITMs are incorporated into progeny HIV and SIV virions and that IFITM expression in infected cells reduces the infectivity of progeny virions (19, 20). The negative impact on infectivity might be due to IFITM interactions with Env, which result in reduced Env processing and incorporation into virions (21). However, the HIV-1 and SIV sensitivity to IFITM is isolate specific and the reasons why some isolates are efficiently inhibited while others are not are unknown (17, 21).

Here, we addressed the question of whether determinants other than the Env protein could impact sensitivity of viral entry to inhibition by IFITM proteins. Such a scenario might account, at least in part, for the strain-specific differences in IFITM sensitivity discussed above and might explain why IFITM sensitivity of HIV/SIV entry was not universally observed. For this, we used previously described vector systems that allow for sensitive detection of viral entry and for robust and comparable expression of IFITM proteins in transduced cells (22, 23). We report that the virion context in which viral envelope proteins are presented as well as the efficiency of Env incorporation into particles can impact sensitivity to IFITM, suggesting that the determinants controlling inhibition of viral entry by IFITMs are more complex than initially appreciated.

## RESULTS AND DISCUSSION

### The efficiency of IFITM-mediated entry inhibition depends on the viral vector.

We first asked whether the nature of the virion on which a viral glycoprotein is presented can impact IFITM-dependent entry inhibition. To address this question, we compared IFITM-mediated inhibition of entry driven by murine leukemia virus Env (MLV-Env), simian immunodeficiency virus Env (SIV-Env), and FLUAV hemagglutinin/neuraminidase (FLUAV-HA/NA) presented in the context of retrovirus (MLV, SIV)- and rhabdovirus (vesicular stomatitis virus [VSV])-based vectors. For these experiments, 293T target cells previously transduced to express IFITMs or chloramphenicol acetyltransferase (CAT) as a negative control were chosen as targets for transduction. For analysis of SIV-Env-dependent transduction, target cells were additionally transfected to express rhesus macaque CD4 and CCR5.

**FIG 1:**
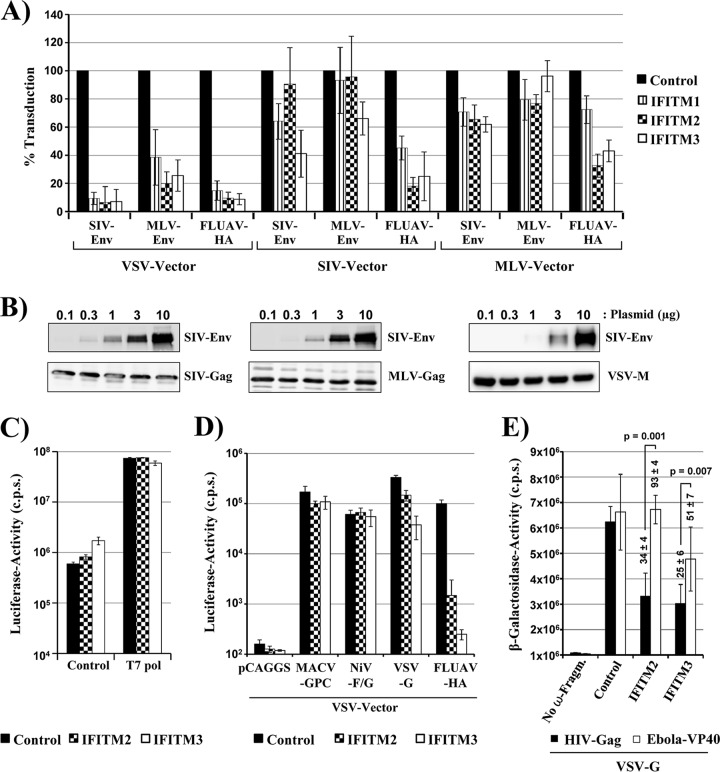
The IFITM sensitivity of viral entry depends on the vector. (A) 293T cells were transduced to express the indicated IFITMs or CAT as a control. Subsequently, the cells were transduced with SIV, MLV, and VSV vectors encoding luciferase and bearing the indicated viral glycoproteins. The average from two to four independent experiments performed with triplicate samples is shown. Transduction of control cells was set as 100%. Error bars indicate standard errors of the means (SEM). In representative experiments, the following luciferase activities were measured in lysates of control cells, which were transduced with the following vectors (values are in counts per second ± standard deviations [SD]). SIV vector pseudotyped with SIV-Env, 393,525 ± 15,044; MLV-Env, 448,071 ± 7,943; FLUAV-HA, 687,410 ± 36,187. MLV vector pseudotyped with SIV-Env, 162,819 ± 44,187; MLV-Env, 460,600 ± 12,781; FLUAV-HA, 42,562 ± 6,365. VSV vector pseudotyped with SIV-Env, 1,170,712 ± 398,070; MLV-Env, 1,240,893 ± 319,341, FLUAV-HA, 1,921,728 ± 269,647. (B) The indicated amounts of SIV-Env-encoding plasmid were transfected into cells employed for production of SIV, MLV, and VSV vectors. Culture supernatants were collected, concentrated by centrifugation through a sucrose cushion and analyzed for SIV-Env, MLV-Gag, SIV-Gag, and VSV-M proteins by Western blotting, employing antibodies raised against these proteins. Similar results were obtained in a separate experiment. (C) Plasmids encoding a VSV minigenome and viral N, P, and L proteins were transfected into 293T cells stably expressing IFITM2, IFTIM3, or control cells in the presence and absence of a T7-polymerase-encoding plasmid. At 48 h posttransfection, cells were lysed and luciferase activities in cell lysates were determined. The results of a single experiment conducted with triplicate samples are shown and were confirmed in a separate experiment. Error bars indicate SD. (D) VSV vectors encoding luciferase and harboring the indicated viral glycoproteins or no glycoprotein (pCAGGS) were inoculated onto 293T cells stably expressing IFITM2 or -3 or CAT (control). The results of a single experiment conducted with quadruplicate samples are shown and were confirmed in two separate experiments. Errors bar indicate SD. (E) Virus-like particles based on HIV-1 p55 Gag (black bars) and Ebola virus VP40 (white bars) fused to the α fragment of β-galactosidase and harboring VSV-G were added to cells stably expressing CAT or the indicated IFITM proteins and transiently expressing the ω fragment of β-galactosidase. Entry efficiency was determined by quantifying β-galactosidase levels in cell lysates. The results of a single experiment performed with triplicate samples are shown. Error bars indicate SD. Similar results were obtained in two separate experiments. Numbers above bars indicate the averages from three independent experiments for which transduction of control cells was set as 100%. Statistical analysis was carried out for normalized data.

Entry of MLV and SIV vectors pseudotyped with FLUAV-HA/NA was reduced by 60 to 80% upon expression of IFITM2 and IFITM3, while MLV-Env-driven entry was barely inhibited by IFITM proteins, as expected (Fig. 1A). Similarly, entry of retroviral particles bearing SIV-Env was only modestly reduced by IFITM proteins. In contrast, expression of all IFITM proteins reduced entry of VSV vectors bearing MLV-Env, SIV-Env, or FLUAV-HA by at least 60% (MLV-Env) or 80% (SIV-Env, FLUAV-HA) (Fig. 1A) and the relative susceptibility to inhibition by IFITM proteins was not dependent on the amount of particles used for transduction (not shown). These results raised the question of whether Env proteins presented on VSV vectors are generally more susceptible to inhibition by IFITM proteins or whether other factors accounted for the differential inhibition. For instance, the reduced IFITM sensitivity of MLV and SIV vectors relative to VSV vectors could be due to reduced glycoprotein incorporation and/or generally diminished entry efficiency of the latter, which might increase IFITM sensitivity. However, Western blot analysis of SIV-Env incorporation into MLV, SIV, and VSV particles did not point toward major differences (Fig. 1B), although the ratio of gp120 to gp160 was somewhat higher for MLV than for SIV/VSV particles (not shown) and a comparison of the luciferase values measured in lysates of transduced cells revealed that relative sensitivity to IFITM was not linked to entry efficiency (not shown). Alternatively, gene expression from VSV but not MLV or SIV vectors might be inhibited by IFITMs. To address this, we used a VSV minireplicon system dependent on the expression of T7 polymerase and cells stably expressing IFITM2 or -3, in which antiviral activity of IFITMs can be more readily visualized than in transduced cells. Transfection of 293T control cells and cells stably expressing IFITM2 or IFITM3 with plasmids encoding the minireplicon system yielded comparable signals (Fig. 1C), indicating that VSV gene expression was not inhibited by IFITMs. Moreover, transduction of these cells by VSV vectors bearing Machupo virus glycoprotein (MACV-GPC), which is known to be IFITM insensitive (6), was only slightly inhibited by IFITMs, and similar results were obtained for a vector bearing the Nipah virus glycoproteins (NiV-G/F) (Fig. 1D), in keeping with the concept that IFITMs do not block VSV gene expression. In contrast, transduction mediated by FLUAV-HA was strongly blocked by IFITM2 and IFITM3 expression (Fig. 1D), confirming that the IFITM proteins exerted antiviral activity in the cell lines tested. Collectively, these results demonstrate that IFITMs do not interfere with VSV gene expression, a finding compatible with a previous study examining the sensitivity of intact VSV to blockade by IFITM proteins and tetherin (24). As a consequence, one must postulate that the particle context in which a viral glycoprotein is presented can impact sensitivity to IFITM, with VSV-based vectors being associated with higher IFITM sensitivity than retroviral vectors.

We next used a simple, two-component system to further explore the potential impact of the virion background on IFITM sensitivity. For this, particles based on HIV Gag and Ebola virus (EBOV) VP40 proteins fused to the α fragment of β-galactosidase were pseudotyped with VSV-G and used to transduce 293T cells stably expressing IFITM2 or -3. In addition, the target cells were transfected to express the ω fragment of β-galactosidase. VSV-G was chosen for pseudotyping of the Gag and VP40 particles, because only this glycoprotein (and not, for instance, EBOV glycoprotein [EBOV-GP] or HIV-Env) consistently yielded signals 30- to 50-fold over background. VSV-G-driven entry was previously shown to be modestly inhibited by IFITM2 and IFITM3 (6), a finding confirmed in the present study with a VSV vector (Fig. 1D). In keeping with these findings, VSV-G-driven entry of Gag particles into cells expressing IFITM2 or -3 was reduced by up to 70% relative to entry into control cells (Fig. 1E). In contrast, IFITM2 expression had no appreciable impact on VSV-G-driven entry of VP40 particles and blockade of entry by IFITM3 was less efficient than that measured for Gag particles (Fig. 1E). These results provide further evidence that the efficiency of the inhibition of viral entry by IFITMs can depend on the nature of the virions presenting the entry-mediating viral glycoproteins.

### IFITM sensitivity of SIV-Env-driven entry depends on the efficiency of Env incorporation into virions.

**FIG 2:**
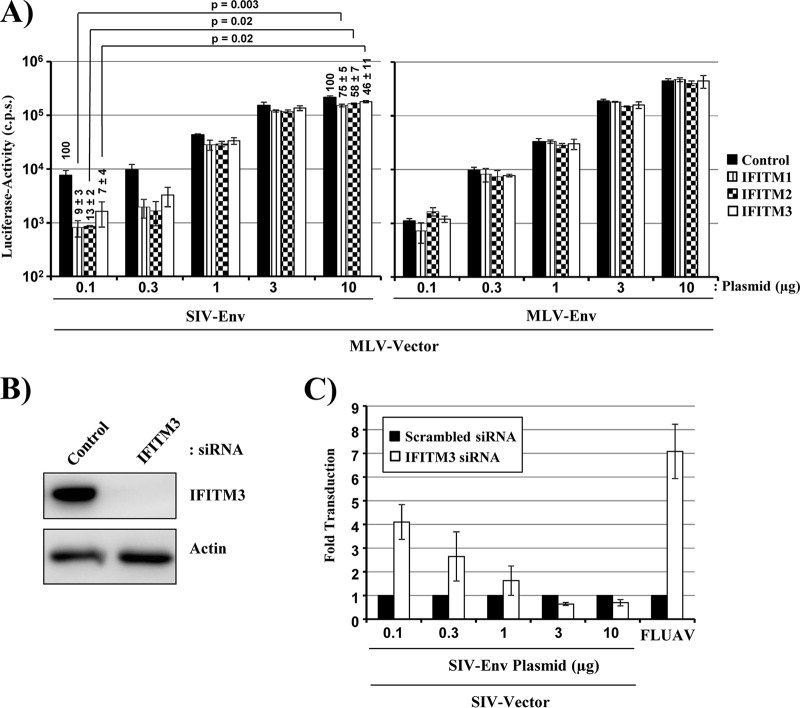
The efficiency of SIV-Env incorporation into virions determines IFITM sensitivity. (A) MLV vectors encoding luciferase and bearing escalating amounts of SIV-Env or MLV-Env were produced by transient transfection of 293T cells with equal amounts of vector plasmid and the indicated amounts of Env-encoding plasmids. Equal volumes of the vector preparations were then inoculated onto 293T cells previously transduced to express IFITMs, and luciferase expression in cell lysates was analyzed at 72 h postransduction. The results of a single representative experiment conducted with triplicate samples are shown. Error bars indicate SD. The results were confirmed in three separate experiments. Numbers above bars indicate the averages from four independent experiments for which transduction of control cells was set as 100%. Statistical analysis was carried out for normalized data. (B) sMAGI cells were transfected with the indicated siRNAs, and IFITM3 expression was analyzed by an immunoblot assay employing an IFITM3-specific antibody. Similar results were obtained in two separate experiments. (C) sMAGI cells transfected as described for panel B were transduced with MLV vectors bearing escalating amounts of SIV-Env, and transduction efficiency was determined as described for panel A. In addition, the cells were infected with FLUAV encoding Gaussia luciferase, and luciferase expression in culture supernatants was analyzed at 48 h postinfection. The average from three independent experiments (two for FLUAV) is shown. Error bars indicate SEM.

The observations that SIV variants can differ in the efficiency of Env incorporation into virions (25, 26) and in sensitivity to IFITM (17) stimulated us to investigate whether these two processes are linked. To answer this question, we produced MLV particles in cells transfected with increasing amounts of SIV-Env- or MLV-Env-encoding plasmids, employing the conditions described for Fig. 1B. The differences in the amount of Env incorporated into virions had no effect on IFITM sensitivity of MLV-Env-driven entry (Fig. 2A). Similarly, we had previously observed that the efficiency of virion incorporation of the EBOV-GP does not alter sensitivity to IFITM (27). In contrast, reducing the amount of SIV-Env in MLV particles markedly increased IFITM sensitivity in a concentration-dependent manner (Fig. 2A). This effect was confirmed in the rhesus macaque-derived sMAGI (simian multinuclear activation of a galactosidase indicator) cell line (28), which expresses endogenous IFITM3 (Fig. 2B). Small interfering RNA (siRNA)-mediated knockdown of IFITM3 expression (Fig. 2B) markedly increased FLUAV infection, as expected, and augmented the efficiency of SIV-Env-mediated transduction (Fig. 2C). The latter effect was inversely correlated with the efficiency of virion incorporation of SIV-Env (Fig. 2C), suggesting that the number of SIV-Env trimers present on the virion surface can impact sensitivity to IFITM.

The antiviral activity of IFITM3 plays an important role in the defense against influenza and potentially other viral diseases (8, 29). It is thus of significant interest to determine the full range of viruses susceptible to inhibition by IFITMs. It is well established that IFITMs target host cell entry (6), and several studies employed vector systems to determine whether viral entry is blocked by IFITM expression (6, 7, 22, 27). We observed that SIV-Env- and MLV-Env-driven entry is IFITM sensitive when these glycoproteins are presented in the context of VSV, but not MLV or SIV, particles. These findings were not due to differential transduction efficiency or major differences in particle incorporation of Env, although it should be pointed out that Gag/M-protein levels cannot be directly compared and that minor differences in particle incorporation efficiency might have remained undetected. Similarly, these observations were not due to a differential impact of IFITMs on retroviral or rhabdoviral gene expression, which were both unaffected (Fig. 1C and data not shown). Notably, virus-like particles (spherical) based on HIV-p55Gag and harboring VSV-G exhibited a higher sensitivity toward inhibition by IFITM2 and -3 than the corresponding particles based on EBOV VP40 (filamentous), and no appreciable differences in particle incorporation of VSV-G were observed (not shown). Therefore, one must postulate that sensitivity of viral glycoproteins to IFITMs can depend on the virion context. It can be speculated that virions of different shapes might exhibit a different propensity to be taken up into the cell via certain uptake mechanisms and thus to be exposed to IFITM proteins. As a consequence, studies with viral vector systems must be complemented by examination of authentic viruses to draw firm conclusions regarding IFITM sensitivity.

The efficiency of IFITM-mediated inhibition of HIV and SIV entry into target cells is isolate dependent (17), for at present unclear reasons. We found that the efficiency of SIV-Env incorporation into the envelope of MLV vectors determines the degree of sensitivity to IFITM. This finding suggests that differences in Env incorporation could contribute to the differential IFITM sensitivity of SIV-isolates. In contrast, the amount of MLV-Env (present study) or EBOV-GP (27) inserted into particles does not modulate IFITM sensitivity. These discrepant observations might be accounted for by differential inhibition of these glycoproteins by IFITMs. Thus, it has been documented that amphotericin B treatment rescues SIV but not EBOV-GP-mediated entry from blockade by IFITMs (17, 27), demonstrating that IFITMs can indeed interfere with viral entry via different mechanisms. Moreover, the recent demonstration that Env and IFITMs interact in transfected cells (shown for HIV-1 Env in reference 21) raises the question of whether such interactions also occur during entry and might contribute to the IFITM-mediated entry blockade. In this case, increasing the amount of SIV-Env in the particle envelope might allow the Env to surpass in titer the IFITM molecules available to inhibit viral entry.

In sum, we identified novel parameters that can impact the sensitivity of viral entry to IFITMs. Our findings should facilitate endeavors to fully define the range of viruses targeted by IFITMs and could help to understand how viruses might evade the antiviral activity of these proteins.

## MATERIALS AND METHODS

### Cell lines and viruses.

Human embryonal kidney (HEK) 293T cells were grown in Dulbecco's modified Eagle's medium (DMEM) supplemented with 10% fetal calf serum (FCS), l-glutamine, and penicillin-streptomycin in a humidified atmosphere containing 5% CO_2_. 293T cells stably expressing IFITM proteins or CAT (22) as well as CMMT-CD4-LTR-β-Gal (sMAGI, simian multinuclear activation of a galactosidase indicator) cells were previously described (28) and were also grown in DMEM supplemented with FCS and antibiotics. The BHK-G43 cell line, which expresses VSV-G upon mifepristone treatment (kindly provided by Georg Herrler), has also been described previously (30). Further, a previously reported FLUAV encoding Gaussia luciferase was used (31).

### Plasmids.

Plasmids encoding the glycoproteins of murine leukemia virus (MLV-Env) (32), FLUAV (strain A/WSN/33, FLUAV-HA; neuraminidase was coexpressed during particle production to ensure efficient particle release) (33), vesicular stomatitis virus (VSV-G) (34), Nipah virus (NiV-F, NiV-G) (35), Machupo virus (MACV-GPC) (6), and SIVmac239 (SIV-Env) (23) have been described previously. The MLV-based vector pQCXIP encoding IFITM proteins or CAT and the MLV gag-pol-encoding plasmid were previously described (22) and were employed for expression of IFITM proteins in 293T cells. Vector MLV-luc (22) and the SIV-based vector SIVmac239 Δenv Δnef Luc (23), both encoding firefly luciferase (fLuc), were also previously reported and were used to quantify transduction mediated by the viral glycoproteins under study. The plasmids encoding EBOV VP40 and HIV-1 p55 Gag fused with the α fragment of β-galactosidase and the plasmid encoding the ω fragment of β-galactosidase have also been documented previously (36, 37). A VSV minigenome (VSV-mini) was constructed as follows. First, the genetic information for all VSV genes and enhanced green fluorescent protein (eGFP) was excised from the pUC18_VSV24* plasmid, a modified VSV genome in which each gene is flanked by identical restriction sites for convenient cloning (kindly provided by Gert Zimmer), making use of AvrII and NheI restriction sites of the nucleoprotein and RNA-dependent RNA polymerase open reading frames (ORFs), respectively. By this process, only the leader and trailer sequences of the parental VSV genome were left between the T7 promoter (T7Pro) at the 5′ end and a hepatitis delta virus ribozyme (HDV-R) and the T7 terminator (T7Ter) at the 3′ end. Next, a chimeric reporter gene consisting of eGFP and fLuc (eGFP-fLuc), fused via a linker sequence (GGG CCC GAT CCT CCT GTT GCT ACT), was generated by overlap extension PCR and ligated between the leader and trailer sequences, yielding a VSV minigenome of positive orientation (5′-T7Pro-leader-eGFP-fLuc-trailer-HDV-R-T7Ter −3′). To generate expression plasmids for VSV-N, -P, and -L, which together build the viral polymerase complex responsible for genome replication and synthesis of subgenomic mRNAs, the respective ORFs from the pUC18_VSV24* plasmid were amplified by PCR and inserted into the pCAGGS vector by restriction digest (VSV-N, EcoRI/NheI; VSV-P, EcoRI/NheI; VSV-L, NheI/NheI) and ligation. All PCR-amplified sequences were verified by automated sequencing.

### Production of retroviral vectors and transduction experiments.

The production of retroviral vectors encoding IFITM proteins or fLuc and pseudotyped with a viral glycoprotein was described previously (22, 32). In brief, for production of vectors encoding IFITM proteins, 293T cells were cotransfected with plasmids encoding MLV gag-pol and VSV-G and with pQCXIP coding for IFITM proteins or CAT as control. For production of MLV reporter particles, 293T cells were transfected with plasmids encoding MLV gag-pol and the viral glycoprotein under study and an MLV vector coding for fLuc. Similarly, SIV particles were produced by cotransfection of the proviral plasmid SIVmac239 Δenv Δnef Luc containing fLuc in the place of the nef gene and a plasmid encoding the glycoprotein of interest. The culture medium was exchanged at 6 h posttransfection, and supernatants were harvested at 48 h posttransfection. Supernatants were sterile filtered through a 0.45-μm filter, aliquoted, and stored at −80°C. Preparations of luciferase-encoding vectors were normalized for comparable transduction of 293T cells before usage in experiments.

### Production of VSV pseudotypes.

For production of VSV particles, we employed a replication-deficient VSV vector, VSV*ΔG-fLuc, that contains two separate ORFs, coding for eGFP and fLuc, instead of the genetic information for VSV-G (38–40), which was propagated in a previously described VSV-G-expressing, transgenic cell line (BHK-G43 [30]). Briefly, HEK-293T cells were transfected by calcium phosphate precipitation with expression plasmids encoding the viral surface proteins under study. At 16 h posttransfection, the cells were inoculated with VSV*ΔG-Luc at a multiplicity of infection (MOI) of 3 for 1 h at 37°C and 5% CO_2_. Subsequently, the cells were washed and finally received fresh culture medium supplemented with (MACV-GPC, NiV-F/G, FLUAV-HA/NA, MLV-Env, and SIV-Env pseudotyped vectors) or without (VSV-G pseudotyped vectors) neutralizing antibodies against VSV-G (I1; hybridoma supernatant from ATCC CRL-2700). After an additional incubation period of 16 to 20 h, VSV pseudoparticle-containing supernatants were collected, clarified from cell debris by centrifugation, and aliquoted. Aliquots were stored at 4°C for a maximum of 7 days.

### Inhibition of viral entry by IFITM proteins.

In order to analyze IFITM-mediated inhibition of transduction by retroviral vectors, 293T cells were seeded at a density of 10^4^ cells per well in 96-well plates and then spinoculated (41) at 4,000 × *g* for 30 min with IFITM- or CAT-encoding vectors. Alternatively, 293T cells stably expressing IFITM proteins or CAT were used as targets. After incubating the cells for 48 h at 37°C, the culture supernatants were replaced by 50 μl of fresh culture medium. Subsequently, the cells were inoculated with 50 μl of luciferase-normalized vectors harboring the viral glycoproteins under study and incubated for 8 h. Thereafter, the supernatants were replaced by 150 μl of fresh culture medium, and fLuc activity in cell lysates was measured at 72 h postransduction. For this, the cell culture supernatants were removed and the cells were washed with phosphate-buffered saline (PBS). Next, 50 μl of 1× luciferase cell culture lysis reagent (Promega) in PBS was added to each well, and the wells were incubated for 30 min at room temperature before the cell lysate was transferred to a white, opaque-walled 96-well plate (Thermo Scientific). The measurement of the fLuc activity was carried out in a microplate reader, Plate Chameleon V (Hidex), using the MicroWin2000 software (version 4.44; Mikrotek Laborsysteme GmbH) and fLuc substrates from the commercial luciferase assay system (Promega) or Beetle-Juice (PJK) kits. Transduction efficiency, represented by fLuc activity, was displayed either in counts per second (c.p.s.) or as normalized values. For analysis of IFITM-mediated inhibition of transduction by rhabdoviral vectors, target cells were prepared as described above and inoculated with VSV pseudotypes for 1 h at 37°C and 5% CO_2_. Afterwards, the cells were washed and further incubated with fresh culture medium for 16 to 18 h followed by quantification of luciferase activity in cell lysates as described above.

### Analysis of VSV genome replication.

To assess VSV genome replication, plasmid VSV-mini was transfected along with pCAGGS plasmids encoding VSV-N, VSV-P, VSV-L, and T7 polymerase (kindly provided by Andrea Marzi) into 293T cell lines stably expressing IFITM proteins or CAT via calcium phosphate precipitation. Upon transfection, the T7 polymerase synthesizes a cRNA copy of the minigenome, which is in the same orientation as authentic VSV genomes in the context of viral infection. The copy RNA then serves as a template for genome replication and synthesis of subgenomic mRNA, which is dependent on VSV-N, -P, and -L. However, instead of the synthesis of mRNAs for viral proteins, only mRNA for eGFP-fLuc is synthesized. At 48 h posttransfection, the medium was removed and fLuc activity in cell lysates was quantified as described above.

### siRNA knockdown in sMAGI cells.

To knock down IFITM expression in sMAGI cells, 6,000 cells per well were seeded in 96-well plates and then transfected with siRNAs directed against IFITM3 (Santa Cruz) or against a scrambled sequence using Lipofectamine RNAiMax (Invitrogen) transfection reagent, according to the manufacturer's protocol. At 48 h posttransfection, the medium was replaced with fresh DMEM and the cells were transduced with the MLV vector encoding luciferase and pseudotyped with SIV-Env. In parallel, IFITM3 expression was analyzed as described below. Luciferase activities in cell lysates were analyzed at 72 h posttransduction as described above. In parallel, cells were inoculated with a Gaussia luciferase-encoding influenza virus at an MOI of 10, input virus was removed by washing, and luciferase activities in cell supernatants were determined at 48 h postinfection.

### Immunoblotting.

For analysis of SIV-Env and Gag or VSV-M incorporation into retro- and rhabdoviral particles, respectively, particles were purified via ultracentrifugation through a 20% sucrose cushion. The pellets were resuspended in SDS-PAGE sample buffer and analyzed by Western blotting. MLV-Gag was detected using an anti-MLV-p30 mouse antibody (Acris). SIV-Gag was detected using an anti-SIV-p27 mouse antibody (55-2F12). VSV-M detection was performed using an anti-VSV-M antibody (Kerafast). SIV-Env was detected using mouse monoclonal antibody DA6, which recognizes an epitope within gp120 (42). A horseradish peroxidase (HRP)-coupled goat anti-mouse IgG (H+L) antibody (Dianova) was used for detection employing a commercially available kit (Amersham). To analyze IFITM expression in sMAGI cells, an IFITM3-specific antibody was used (Proteintech).
